# Effects of Elevated Temperature on the Residual Behavior of Concrete Containing Marble Dust and Foundry Sand

**DOI:** 10.3390/ma15103632

**Published:** 2022-05-19

**Authors:** Aditya Kumar Tiwary, Sandeep Singh, Raman Kumar, Jasgurpreet Singh Chohan, Shubham Sharma, Jujhar Singh, Changhe Li, R. A. Ilyas, M. R. M. Asyraf, Mohammad Abdul Malik

**Affiliations:** 1 Department of Civil Engineering, Chandigarh University, Mohali 140413, India; adadtiwary15@gmail.com (A.K.T.); drsandeep1786@gmail.com (S.S.); 2 University Centre of Research and Development, Mechanical Engineering Department, Chandigarh University, Mohali 140413, India; ramankakkar@gmail.com (R.K.); jaskhera@gmail.com (J.S.C.); 3 Department of Mechanical Engineering, IK Gujral Punjab Technical University, Main Campus, Kapurthala 144603, India; jujharsingh2085@gmail.com; 4 School of Mechanical and Automotive Engineering, Qingdao University of Technology, Qingdao 266520, China; sy_lichanghe@163.com; 5 School of Chemical and Energy Engineering, Faculty of Engineering, Universiti Teknologi Malaysia, Johor Bahru 81310, Malaysia; ahmadilyas@utm.my; 6 Centre for Advanced Composite Materials, Universiti Teknologi Malaysia, Johor Bahru 81310, Malaysia; 7 Institute of Energy Infrastructure, Universiti Tenaga Nasional, Jalan Ikram-Uniten, Kajang 43000, Malaysia; asyrafriz96@gmail.com; 8 Engineering Management Department, College of Engineering, Prince Sultan University, Riyadh 11586, Saudi Arabia

**Keywords:** marble dust, foundry sand, residual properties of concrete, elevated temperature, annealing, quenching

## Abstract

Concrete is a composite material that is commonly used in the construction industry. It will certainly be exposed to fires of varying intensities when used in buildings and industries. The major goal of this article was to look into the influence of mineral additions such as foundry sand and marble dust on the residual characteristics of concrete. To examine the behavior of residual characteristics of concrete after fire exposure, marble dust was substituted for cement and fine sand was substituted for foundry sand in varying amounts ranging from 0% to 20%. It aided in the better disposal of waste material so that it might be used as an addition. The purpose of the experiment was to see how increased temperatures affected residual properties of concrete, including flexural strength, compressive strength, tensile strength, static as well as dynamic elastic modulus, water absorption, mass loss, and ultrasonic pulse velocity. At temperatures of 200 °C, 400 °C, 600 °C, 800 °C, and 1000 °C, the typical fire exposure behavior of concrete was investigated. The effects of two cooling techniques, annealing and quenching, on the residual properties of concrete after exposure to high temperatures were investigated in this study. Replacement of up to 10% of the cement with marble dust and fine sand with foundry sand when concrete is exposed to temperatures up to 400 °C does not influence the behavior of concrete. At temperatures above 400 °C, however, the breakdown of concrete, which includes marble dust and foundry sand, causes a rapid deterioration in the residual properties of concrete, primarily for replacement of more than 10%.

## 1. Introduction

The inhabitants of a structure must adhere to certain fire safety standards stated in the building regulations. Several kinds of research on concrete’s behavior under fire have been carried out. The results show that when concrete is subjected to fire at high temperatures, the various properties of concrete reduce or are affected negatively. Some of such properties of concrete include tensile strength, elastic modulus, compressive strength, and many others [1,2,3,4]. There are various other elements, including heating rate, aggregate, specimen size, moisture content, specimen age, and so on, that influence the change in mechanical characteristics. For manufacturing high-density blocks, the primary need is to produce high-density concrete blocks. Such blocks are formed from cast concrete using Portland cement and aggregate, often sand and fine gravel [5,6,7].

As per many incidences of fire in India, the temperature during the fire was observed up to 800 °C [8]. The high number of deaths and material loss caused by fires is largely due to the poor condition of fire services and negligent application of standards of fire safety in factories and buildings (IRS 2017). The significant loss of strength in high-strength concrete occurs when the temperature is around 100 °C (on initiation of water removal process) as well as 200 °C, while absorbed water and moisture spread to the concrete section’s outer layer; therefore, internal forces are affected through the entire specimen as well as its strength being reduced; therefore, when the concrete’s original strength is high, it results in greater strength losses.

Yuksel et al. [8] examined the strength properties and it was observed that the mass loss characteristics are affected by high temperatures when bottom ash or blast-furnace slag was utilized as a fine material in concrete. For a high range of temperatures, the bottom ash or slag replacement ratio was found to have little influence on mass loss. Moreover, it was established in the study that for all replacement ratios, the rate of decrease in compressive strength of these concrete samples owing to elevated temperature was found to be greater than the control mix. Numerous substitutes have been studied in recent decades to replace traditional resources of concrete manufacturing. Some of the most commonly adopted substitutes are steel slag, brick waste, recycled aggregates, and dimension stone waste [9,10,11,12,13]. The studies have focused on analyzing the impacts of rising temperatures on substituted concrete [14,15,16,17,18,19,20,21,22,23,24,25,26]. One such kind of research focused on analyzing the impact of increasing temperature on shredded Polyethylene Terephthalate (PET) aggregates as a fine aggregate replacement [15]. The rate of decrease in flexural strength owing to rising temperatures was found to be increased as replacement levels increased.

Another study was carried out by Poon et al. [16] incorporating silica fume, fly ash, and blast furnace slag to examine the compressive strength of ordinary and high-strength pozzolanic concrete under temperatures up to 800 °C. The concrete incorporating silica fume had abrupt spalling, whereas the concrete containing blast furnace slag and fly ash had a scattered network of local cracks but no spalling. The compressive strength of the pozzolanic concrete was much lower than that of the control mix.

Siddique and Kaur [17] researched concrete containing granulated glass blast furnace slag (GGBFS), and the results showed that when compared to the control mix, compressive strength and mass loss were not significantly different at 100 °C. However, mass loss rose and elastic modulus along with compressive strength dropped dramatically at 350 °C. Another experiment to evaluate the performance of concrete containing metakaolin and fly ash at elevated temperatures was performed by Nadeem et al. [27]. It showed that concrete samples incorporating metakaolin and fly ash had higher chloride permeability, sorptivity, and mass loss than control mixes.

A study carried out by Kore Sudarshan D. and Vyas [28] examined the mechanical characteristics of marble dust-encrusted concrete at temperatures in the range of 200–800 °C. They observed that after being exposed to heat up to 400 °C, concrete mixes produced from marble debris had outstanding mechanical properties. Concrete began to degrade when the temperature increased over this threshold.

The main scope of this study is fire safety to protect people’s lives and property from fire accidents. A fire outbreak has become the third major risk to business continuity and operations, as recorded by the India Risk Survey (Indian Risk Survey report) 2018 [29]. In the year 2018, 13,099 fire accident cases were reported in India, which included a fire explosion in a cracker factory on 4 July 2018, a fire explosion at a BPCL plant on 9 August 2018, a fire accident in Mumbai hospital on 17 December 2018, etc. In 2016, fire outbreaks were placed eighth in rank for being one of the biggest threats to businesses in a report by the IRS. There was an increase of 300% in cases of fire accidents in commercial buildings between 2014 (179 cases) and 2015 (716 cases). Furthermore, in government buildings, fire outbreaks jumped by 218% in the same phase (35 cases in 2015 compared to 11 cases in 2014). The ADSI report describes those residential buildings that are most susceptible to fire outbreaks. In 2015, a total of 7493 fire outbreak cases were reported in residential buildings with an increase of 100% from the previous year, 2014 (3736 cases). In fact, 42% of the casualties happened due to accidental fire in 2015 in residential buildings [30]. Therefore, this study makes an important point to investigate the residual behavior of concrete containing marble dust and waste foundry sand under elevated temperatures.

The main objectives of this study are to investigate the behavior of residual concrete properties such as tensile strength, flexural strength, compressive strength, ultrasonic pulse velocity, static as well as dynamic elastic modulus, and mass loss when exposed to higher temperatures (between 200 °C and 1000 °C). Following the ISO-834 fire curve standards [31], the concrete specimens were heated to the following temperatures: 200°C, 400°C, 600 °C, 800 °C, and 1000 °C. The residual properties of concrete have been investigated using two cooling regimes: annealing and water quenching. The research demonstrated the utilization of marble dust (0–20%) as cement and foundry sand (0–20%) like sand. The effects of the two cooling regimes, annealing and water quenching, at temperatures in the range of 200 °C to 1000 °C have been assessed on the outcomes of replaced and non-replaced concrete specimens.

## 2. Materials and Method

### 2.1. Materials

For the present study, Jaypee Cement’s Ordinary Portland Cement of grade 43 was used. The specific gravity of 3.15 is in line with IS 8112:2013 [32] for this investigation. The different chemical characteristics of cement have been tabulated in Table 1. The present study employed quartz-based fine aggregate that has been produced from natural sand. The maximum grain diameter of the fine aggregate is 4.75 mm and it has a specific gravity of 2.69. Further, basalt-based coarse aggregate with a size range of 10 mm to 20 mm and a specific gravity of 2.74 was used for the investigation. To achieve the necessary workability, a superplasticizer complying with IS 9103:1999 [33] was employed as an admixture.

**Figure 1:**
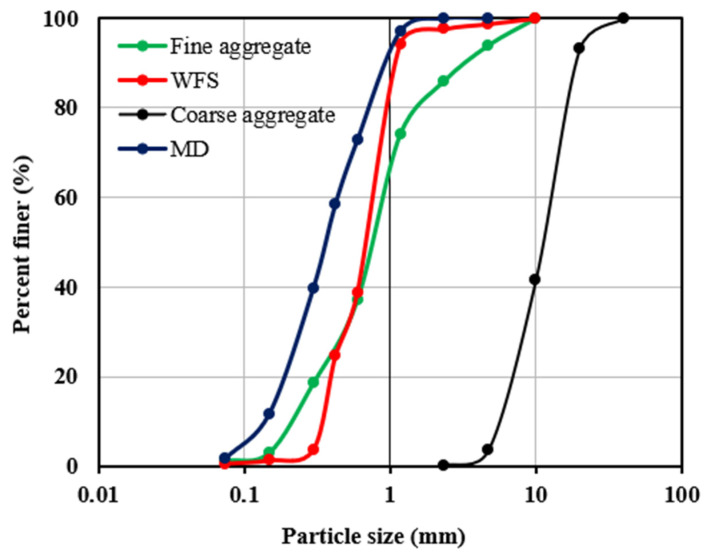
Particle size distribution curve for samples.

For the present investigation, cement has partially been replaced with marble dust. The composition of marble dust has been illustrated in Table 2. It mentions that the marble dust is composed of 40.45% CaO and 28.35% SiO_2_ with a specific gravity of 2.6. In addition to it, the fine aggregates have been partially replaced with foundry sand with a specific gravity of 2.6 and water absorption of 0.68%. The particle size distribution curve for fine aggregate, foundry sand, coarse aggregate, and marble dust is shown in Figure 1. Table 3 shows the chemical composition of foundry sand.

### 2.2. Mix Proportions

For the present investigation the concrete mix was made according to IS 10262:2009 [34] using marble dust as a partial substitute for cement ranging from 0% to 20%, and foundry sand as a partial substitution for sand in the range of 0% to 20%. The ratios of Concrete mix with marble dust and foundry sand were 1:1.3:2.6 which have been used in the present study are given in Table 4. The water-to-cement ratio that was adopted for the experimental work was 0.45. The mixtures were initially dry-mixed in the mixer for 2–3 min. The quantity of super-plasticizer was adjusted to preserve the workability and homogeneity of the mixtures. A table vibrator was used to vibrate the concrete mix in mold once it exhibited the necessary workability for uniform marble dust and foundry sand distribution. The specimens were wrapped with sheets of plastic and maintained at room temperature for 24 h before demolding [35].

### 2.3. Casting of Specimens

The specimen with the specified ratio of Concrete mix with marble dust and foundry sand was developed. To determine the 28th-day compressive strength of prepared samples in accordance with BIS: 516-1999 [36] 150 mm × 150 mm × 150 mm cubes were cast. All the specimens were de-molded after 24 h and cured in a water tank at room temperature until the test’s defined age [36,37]. In order to determine the tensile strength of the concrete, cylindrical specimens were created of size 150 mm × 300 mm in accordance with BIS: 516-1999 [38]. For evaluating the flexural strength of the concrete mix, 100 mm × 100 mm × 500 mm specimens were cast and tested with reference to BIS: 516-1999 [36]. As far as the assessment of homogeneity and quality of concrete is concerned, the ultrasonic pulse velocity test was performed in accordance with BIS 13311 (Part 1): (1999b) [37]. For evaluation and analysis of the concrete mix characteristics, an average of three samples were used.

### 2.4. Heating Regime

**Figure 2:**
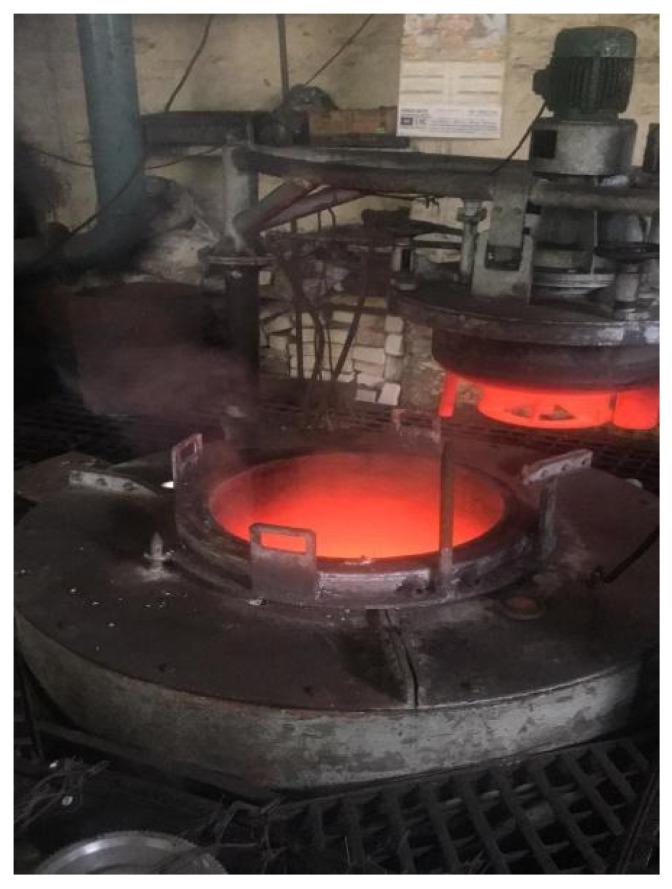
Heating furnace.

**Figure 3:**
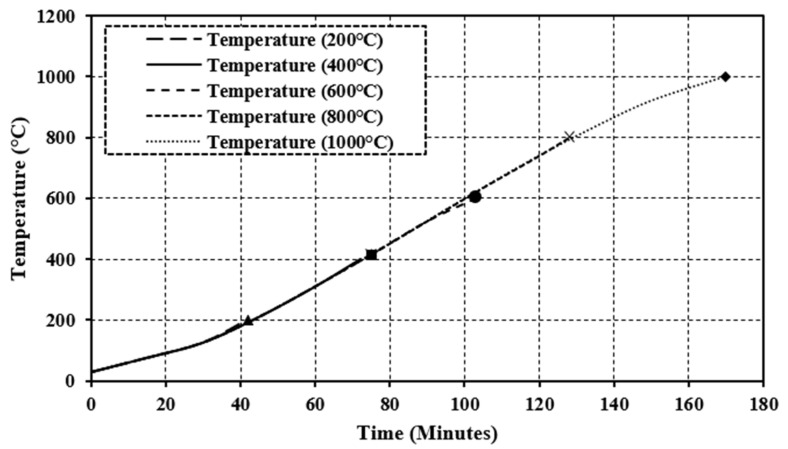
Temperature–time curve for specimens.

**Figure 4:**
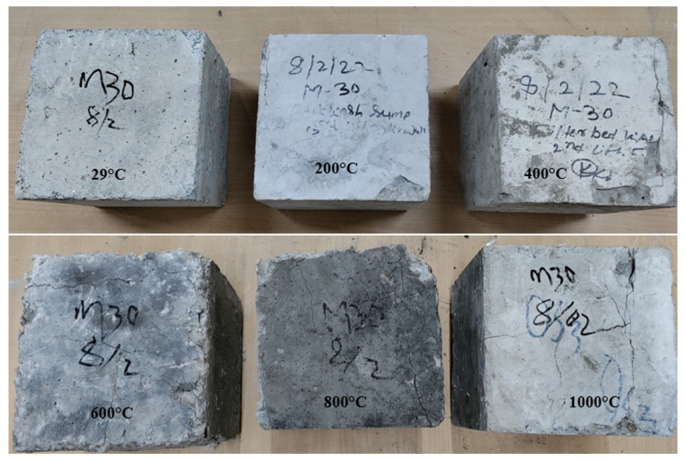
Concrete samples after elevated temperature.

The concrete samples were heated in a furnace at a steel plant in Chandigarh as shown in Figure 2. The industrial furnace had a heating power of 1800 °C. The heating chamber was circular in shape, with a diameter of 2.5 m and a height of 3 m. The prepared concrete specimens were placed in the furnace with the help of a special trolley. After the placement of the specimens, the furnace was closed by using a furnace cap to heat to elevated temperatures. The guidelines provided in the ISO-834 [31] fire curve were followed to heat the specimens. The specimen was heated within the temperature range of 200 °C to 1000 °C. The curves of time–temperature thus formed for heated concrete specimens are shown in Figure 3. To note the temperature reading during the time of exposure, a digital meter was attached to the furnace and the readings for all the specimens were noted at an interval of 15 min. The furnace was turned off immediately as the required temperature was achieved and for the next 24 h, all the specimens were kept in the same furnace to investigate the behavior of concrete at the annealing temperature as heated concrete samples are shown in Figure 4.

### 2.5. Cooling Regimes

Two methods were employed to cool down the specimens: quenching and annealing. The process of annealing was carried out by holding the testing specimens in the furnace for 24 h even after turning off the furnace when the target temperature was reached. In quenching, the specimen was heated in the furnace until the target temperature was reached and was then put into a water bath. A special trolley was used to lift out the heated specimens from the furnace which also helped in immersing the specimens in the water bath for cooling.

### 2.6. Specimens under Raised Temperature

A total of 54 specimens were examined (18 cubes, 18 cylinders, and 18 beams) including three specimens at ambient temperature (29 °C) and other specimens tested after high-temperature exposure ranging from 200–1000 °C. The concrete specimens were heated in a furnace used in the steel industry. The heating rate of specimens increased with time and varied from 5 °C/m to 6 °C/m. Some studies have previously investigated a similar heating rate (4.5 °C/m to 8 °C/m) [38,39]. The curves of time–temperature for heated specimens are shown in Figure 3.

### 2.7. Compressive Strength

The 28th-day compressive strength of samples was evaluated by testing cubes with dimensions 150 mm × 150 mm × 150 mm using a compressive testing machine at a loading rate of 13.7 N/mm^2^/m as per BIS: 516-1999 [36]. At the age of 24 ± 1 h, all of the specimens were de-molded and cured in a water tank at ambient temperature (29 °C) until the test age was reached. Three specimens were examined at room temperature, while the remaining fifteen were tested after elevated temperature exposure.

### 2.8. Flexural Strength

Beam specimens with dimensions of 100 mm × 100 mm × 500 mm were cast to determine the flexural strength as per BIS: 516-1999 [36]. The specimen was supported on the bed of the testing machine by two steel rollers with a diameter of 38 mm, positioned such that the gap between the rollers was 40 mm. Two comparable rollers positioned at the third places of the supporting span spaced at 20 cm center to center applied the load. The load was applied over two lines 20 cm off to the highest region as cast in the mold when the specimens were put in the device. The loading rate was 0.68 N/mm^2^/mm. Three specimens were examined at room temperature, while the remaining fifteen were tested after being exposed to high temperatures.

### 2.9. Tensile Strength

Tests were conducted on 150 mm × 300 mm cylinders using a compression testing machine with a loading rate of 13.7 N/mm^2^/m to determine the 28th-day tensile strength as per BIS: 516-1999 [38]. At the age of 24 ± 1 h, all of the specimens were de-molded and placed in a water tank at ambient temperature (29 °C) for curing till the test age was reached. Three specimens were evaluated at room temperature, while the remaining fifteen specimens were tested after elevated temperature exposure.

### 2.10. Static Elastic Modulus

The elastic modulus was evaluated using an ASTM C469 [40] cylinder specimen with dimensions of 150 mm × 300 mm. Specimens were put through their paces on 300-ton-capacity automated compression testing equipment with lateral extensometer as well as longitudinal compressometer attachments. The specimen’s axis was aligned with the compression testing machine’s center of thrust. The load was applied progressively while the machine moved at a pace of 240 ± 35 kN/m^2^/s. The applied load as well as the associated stresses were measured. The elasticity modulus was then determined using the following equation:

(1) $$E_{s}\;=(\sigma_{2}-\sigma_{1})/(\varepsilon_{2}-\varepsilon_{1})$$

Here, $\sigma_{2}$ is the stress that corresponds to 40% of the ultimate load, $\sigma_{1}$ is the stress that corresponds to a longitudinal strain, $\varepsilon_{1}$ is the actual strain at 0.000050, and $\varepsilon_{2}$ is the longitudinal strain generated by stress 2.

### 2.11. Dynamic Elastic Modulus

The measured Ultrasonic Pulse Velocity was used to compute the dynamic elastic modulus of hardened concrete subjected to elevated temperatures (UPV) as per IS 13311 (Part 1): 1992 [37]. The dynamic elastic modulus was estimated utilizing Topcu and Bilir’s equation [41].

(2) $$E=\frac{\rho \;\left(1+\mu \right)\left(1-2\mu \right)}{\left(1-\mu \right)}\times V^{2}$$

Here *E* = dynamic modulus of elasticity (MPa), *V* = ultrasonic pulse velocity (m/s), *ρ* = density (kg/m^3^) and *μ* = dynamic Poisson’s ratio (0.2 to 0.35).

### 2.12. Water Absorption

A water retention test was performed on concrete specimens including foundry sand and marble dust that had been subjected to fire at varying temperatures as per sub-clause 2.1 of DIN 1045:1988 [42]. For this experiment, cubes of concrete with dimensions of 150 mm × 150 mm × 150 mm were employed. The mold was then filled with concrete associated with the water absorption apparatus and water was added to the water drum to apply pressure of 0.6 N/mm^2^ on the concrete surface. The depth of penetration of the water was reported after 3 days of testing of specimens by compression testing machine.

### 2.13. Mass Loss

Mass loss of the concrete specimen was obtained through cube samples with dimensions 150 mm × 150 mm × 150 mm after exposure to elevated temperature. The loss in mass of each cube specimen was determined by weight measurement of the cube specimen after exposure to an elevated temperature ranging from 200 °C to 1000 °C.

### 2.14. Ultrasonic Pulse Velocity

The ultrasonic pulse velocity of the specimens was recorded once they were exposed to high temperatures of 200 °C, 400 °C, 600 °C, 800 °C, and 1000 °C. Cube specimens were subjected to an ultrasonic pulse velocity test in accordance with IS 13311 (Part 2): 1992 [43] even before the compression test.

## 3. Results and Discussions

### 3.1. Compressive Strength after the Elevated Temperature in Case of Annealing

**Figure 5:**
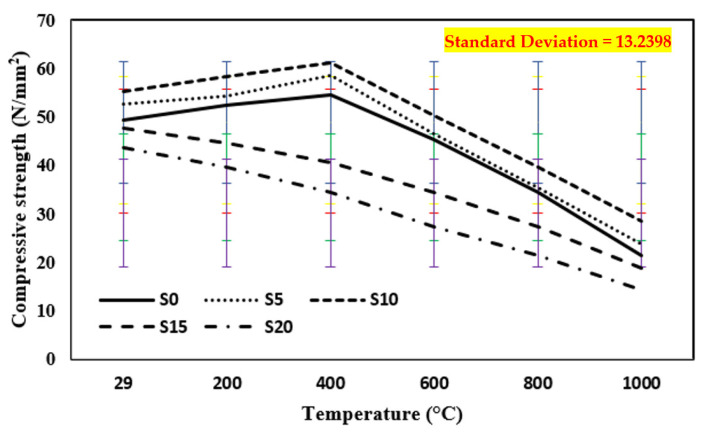
Compressive strength of concrete after exposure to an elevated temperature at annealing. The standard deviation in all curves showing as: red for S0, yellow for S5, blue for S10, green for S15 and purple for S20.

The strength of a control mix and concrete containing marble dust and foundry sand in compression when exposed to higher temperatures in the range 200–1000 °C in the case of annealing is shown in Figure 5. As the temperature was raised from 29 °C to 400 °C, the compressive strength of the control mix and concrete incorporating 10% foundry sand and marble dust each increased. However, further increases in temperature lowered the compressive capacity of the specimens. With the temperature rise, there was also a continual loss in strength with 15% and 20% replacement of both components, i.e., marble dust and foundry sand, respectively [44,45,46,47,48,49,50,51,52,53,54,55,56,57,58,59]. This was caused by the incompatibility between the thermal expansions of both aggregate as well as cement paste for elevated temperatures [8,16,17]. In reality, when the temperature rises, coarse aggregate expands; the C-S-H gel, on the other hand, eliminates both chemically as well as physically bound water, causing shrinkage and fractures to form. Another factor might be the reduction in cohesive forces caused by water expansion at this temperature [44,45]. When the temperature reaches around 400 °C, some aggregates constituting silicates disintegrate, as a result of which the strength is reduced. At ambient temperature (29 °C), the compressive strength of samples increased by 10.7% when marble dust replaced cement by 10% and foundry sand replaced fine sand and decreased by 11% with 20% substitutions of marble dust and foundry sand as cement and fine sand respectively. The compressive strength of concrete samples tested at elevated temperatures increased by 21.5% in the case of annealing up to 400 °C on 10% substitution of marble dust as cement and foundry sand as fine sand and then decreased by 52.5% as temperature increased from 600 °C to 1000 °C. This is related to the creation of tiny fractures in both normal and marble dust and foundry sand concrete on account of the breakdown of C-S-H gel along with the voids as well as empty water capillaries.

### 3.2. Compressive Strength after the Elevated Temperature in Case of Quenching

**Figure 6:**
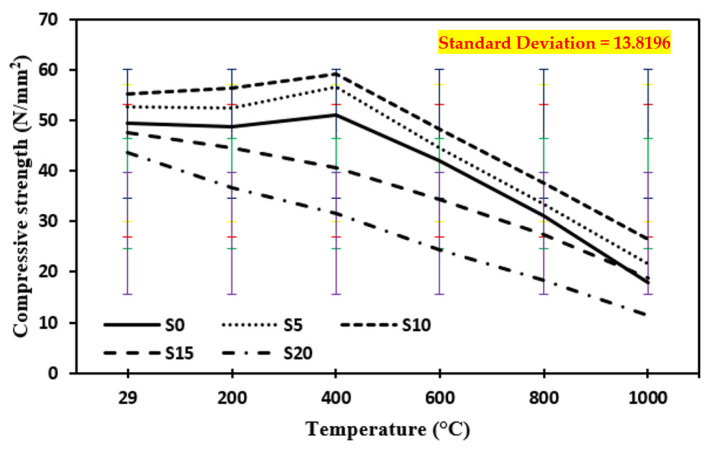
Compressive strength of concrete after exposure to an elevated temperature at quenching. The standard deviation in all curves showing as: red for S0, yellow for S5, blue for S10, green for S15 and purple for S20.

Figure 6 shows the compressive strength of a control mix and concrete containing marble dust and foundry sand, subjected to high temperatures ranging from 200 °C to 1000 °C in the event of quenching. All cases of quenching had a greater loss in compressive strength than the comparable cases of annealing. This might be owing to the thermal shock supplied by water at high temperatures, as well as rapid gas escape, excessive hardness, or surface defects created by unbalanced heating and cooling phases in the case of quenching cooling [28,35,57,58,59,60,61,62,63,64,65,66,67,68,69,70,71,72,73]. The quenching technique creates residual stresses between the concrete’s outer and interior surfaces. This causes tensile strains in the outer portion, which causes the microcracks to grow [44]. Yuzer et al. [45] found that when calcium oxide is quenched, it converts to calcium hydroxide and flows through the pores, causing an increase in volume and significant fractures in concrete. The compressive strength of concrete samples tested at elevated temperatures increased by 16.3% in the case of quenching up to 400 °C elevated temperature on samples with 10% replacement of cement by marble dust and fine sand replaced by foundry sand, and then decreased by 54.8% as temperature increased from 600 °C to 1000 °C. This is related to the creation of tiny fractures in both normal and marble dust and foundry sand concrete owing to the breakdown of C-S-H gel along with the voids and empty water capillaries.

### 3.3. Flexural Strength at Annealing

**Figure 7:**
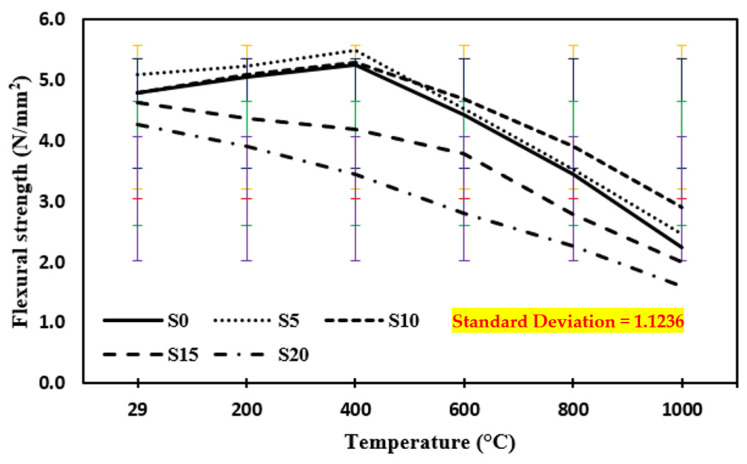
Flexural strength of concrete after exposure to an elevated temperature at annealing. The standard deviation in all curves showing as: red for S0, yellow for S5, blue for S10, green for S15 and purple for S20.

Figure 7 shows the flexural strength of the control mix and concrete including marble dust and foundry sand subjected to higher temperatures of 200 °C, 400 °C, 600 °C, 800 °C, and 1000 °C in the case of annealing. The results show that from a typical ambient temperature of 29 °C to an elevated temperature up to 400 °C, the flexural strength of both concrete mixes rises first. This improvement in flexural strength might be attributed to the fact that following heating, the specimens are cooled via the annealing process, which allows part of the evaporated water to be reclaimed from ambient moisture [35,38,39,72,73,74,75,76,77,78,79,80,81,82,83,84,85,86,87]. When the temperature was raised from 600 °C to 1000 °C, the flexural strengths of both concrete mixes plummeted. The rapid drop in flexural strength is attributed to the development of widening fractures around 1000 °C. The connection between aggregate and cement pastes is weakened by shrinkage produced by the evaporation of free water and chemically bound water from the cement paste. The flexural strength of concrete samples tested at elevated temperatures increased by 12.7% with a 5% replacement of marble dust and foundry sand in the case of annealing up to 400 °C elevated temperature and then decreased by 40% with an increase in temperature ranging from 600 °C to 1000 °C. The rapid drop in flexural strength is attributed to the development of widening fractures around 1000 °C. However, the connection between aggregate and cement pastes is weakened by shrinkage produced by the evaporation of free water and chemically bound water from the cement paste.

### 3.4. Flexural Strength at Quenching

**Figure 8:**
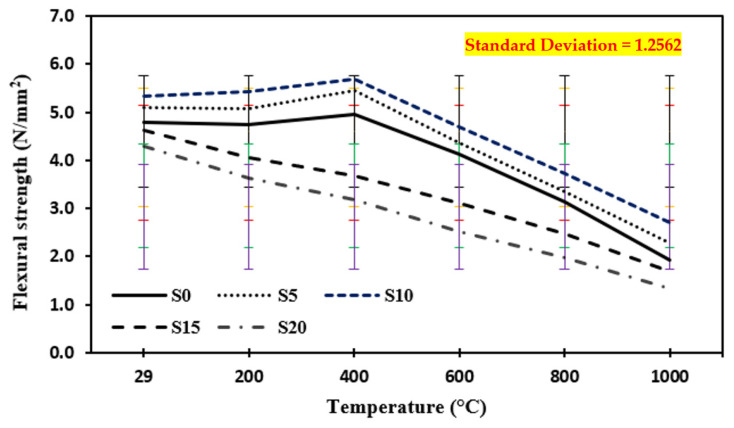
Flexural strength of concrete after exposure to an elevated temperature at quenching. The standard deviation in all curves showing as: red for S0, yellow for S5, blue for S10, green for S15 and purple for S20.

Figure 8 shows the flexural strength of the control mix and marble dust and foundry sand concrete quenched at temperatures of 200 °C, 400 °C, 600 °C, 800 °C, and 1000 °C. The results show that from a typical ambient temperature of 29 °C to an elevated temperature up to 400 °C, the flexural strength of both concrete mixes rises first. The evaporation of free water from the cement paste may be responsible for the rise in flexural strength. When the temperature was raised from 600 °C to 1000 °C, the flexural strengths of both concrete mixes plummeted. The rapid drop in flexural strength is attributed to the development of widening fractures around 1000 °C. The connection between aggregate and cement pastes is weakened by shrinkage produced by the evaporation of free water and chemically bound water from the cement paste [23,24,25,26,84,85,86,87,88,89,90,91,92,93,94,95,96]. The flexural strength of concrete samples tested at elevated temperatures increased by 15.6% with a 10% replacement of marble dust and foundry sand in the case of quenching up to 400 °C elevated temperature and then decreased by 51.7% with an increase in temperature ranging from 600 °C to 1000 °C. The rapid drop in flexural strength is attributed to the development of widening fractures and the sudden release of gases around 1000 °C. However, the connection between aggregate and cement pastes is weakened by shrinkage produced by the evaporation of free water and chemically bound water from the cement paste.

### 3.5. Tensile Strength at Annealing

**Figure 9:**
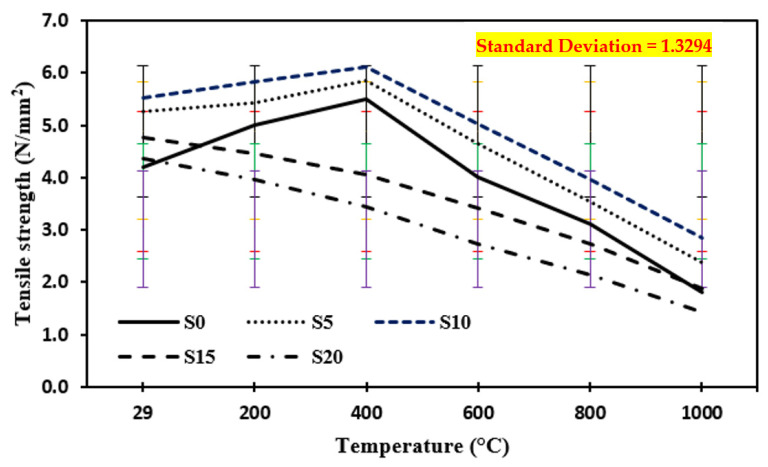
Tensile strength of concrete after exposure to an elevated temperature at annealing. The standard deviation in all curves showing as: red for S0, yellow for S5, blue for S10, green for S15 and purple for S20.

Figure 9 shows the tensile strength of the control mix and concrete including marble dust and foundry sand subjected to higher temperatures of 200 °C, 400 °C, 600 °C, 800 °C, and 1000 °C in the case of annealing. In comparison to the control mix, the tensile strength of concrete containing 10% marble dust and 10% foundry sand was enhanced under higher temperatures up to 400 °C. The tensile strength of concrete samples tested at elevated temperatures increased by 31.1% in the case of annealing up to 400 °C elevated temperature on 10% replacement of cement by marble dust and fine sand replaced by foundry sand and then decreased by 52.5% as the temperature increased from 600 °C to 1000 °C.

### 3.6. Tensile Strength at Quenching

**Figure 10:**
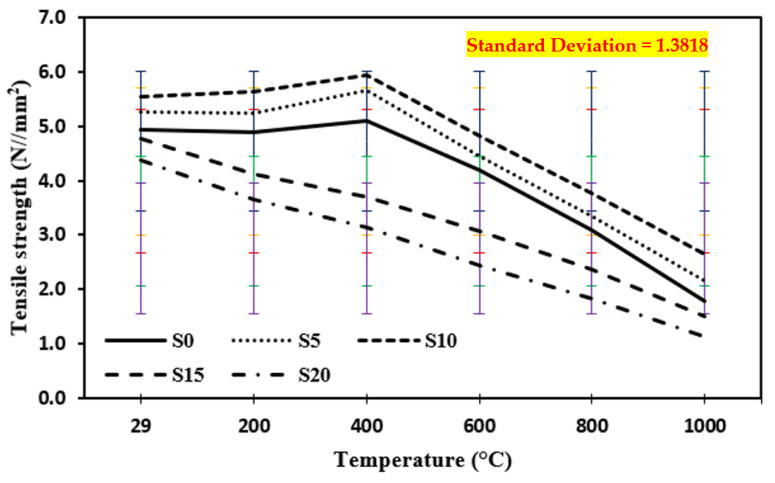
Tensile strength of concrete after exposure to an elevated temperature at quenching. The standard deviation in all curves showing as: red for S0, yellow for S5, blue for S10, green for S15 and purple for S20.

Figure 10 shows the tensile strength of control mix and concrete including marble dust and foundry sand when quenched at temperatures of 200 °C, 400 °C, 600 °C, 800 °C, and 1000 °C. The tensile strength of concrete samples tested at elevated temperatures up to 400 °C increased by 16.9% in the case of quenching on 10% replacement of cement by marble dust and fine sand replaced by foundry sand and then decreased as the temperature increased from 600 °C to 1000 °C by 53.3%.

### 3.7. Static Modulus of Elasticity

**Figure 11:**
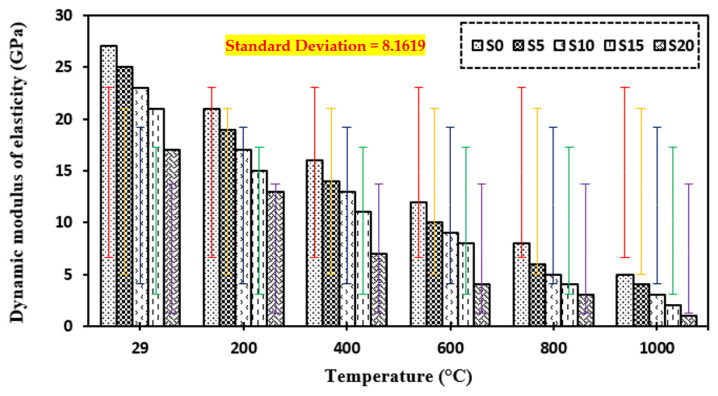
Static modulus of elasticity of concrete after exposure to elevated temperature.

Figure 11 shows the static modulus of elasticity of the control mix and concrete containing marble dust and foundry sand subjected to increased temperatures of 200 °C, 400 °C, 600 °C, 800 °C, and 1000 °C. The static modulus of elasticity was measured to see how the deformation capacity of the concrete mixtures changed over time [17,28,35,39,94,95,96,97,98]. In all cases of concrete exposed to high temperatures, the static modulus of elasticity was shown to be reduced. The percentage reduction in static modulus was increased with elevated temperature; at 1000 °C it was higher for concrete containing marble dust and foundry sand (77.4% for 5% and 86.9% for 20% replacement) than normal concrete (72.7%). The voids that develop as the temperature rises cause the concrete to degrade even more. Furthermore, the loss of static modulus of elasticity is caused by the escape of water vapor and the development of fractures via these gaps.

### 3.8. Dynamic Modulus of Elasticity

**Figure 12:**
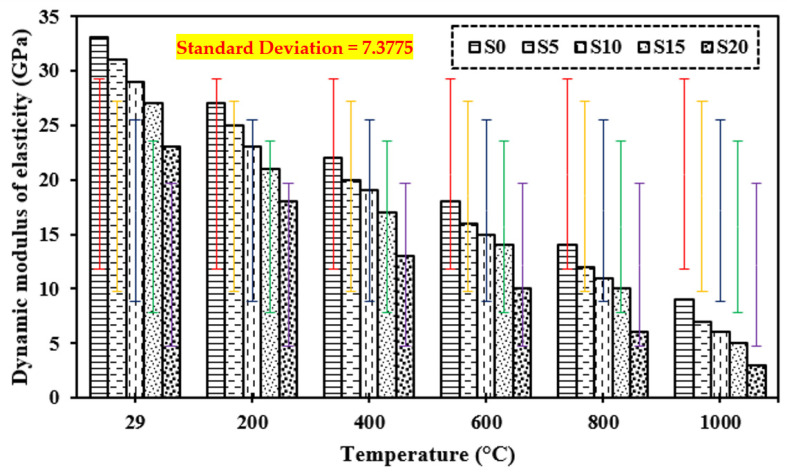
Dynamic modulus of elasticity of concrete after exposure to elevated temperature.

Figure 12 shows the dynamic modulus of elasticity of the control mix and concrete containing marble dust and foundry sand subjected to increased temperatures of 200 °C, 400 °C, 600 °C, 800 °C, and 1000 °C. Elevated temperature causes considerable damage to the cement matrix, according to the dynamic modulus of elasticity [17,28,35,39,94,95,96,97,98,99,100,101,102]. In all cases of concrete exposed to extreme temperatures ranging from 200 °C to 1000 °C, the dynamic modulus of elasticity was shown to be reduced. The percentage reduction for dynamic modulus of elasticity was increased with elevated temperature; at 1000 °C it was higher for concrete containing marble dust and foundry sand (76% for 5% and 94.1% for 20% replacement) than normal concrete (81.5%). The loss of water and voids induced by the applied higher temperature on the specimens causes the dynamic modulus of elasticity to drop.

### 3.9. Water Absorption

**Figure 13:**
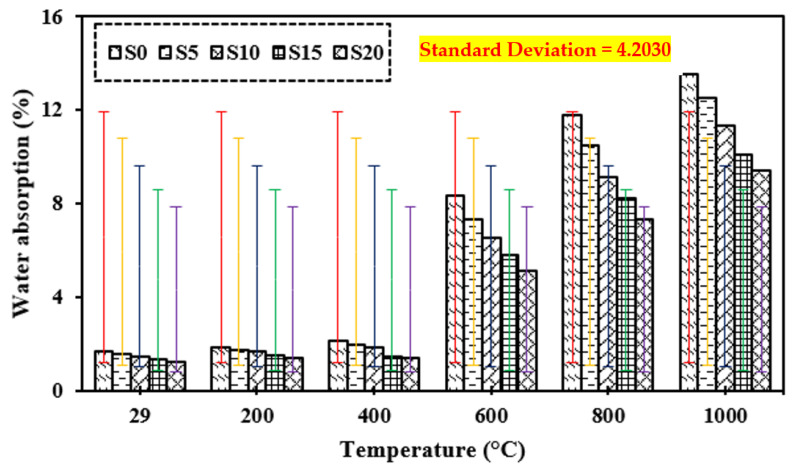
Water absorption of concrete after exposure to elevated temperature.

Figure 13 shows the water absorption of the control mix and concrete including marble dust and foundry sand when subjected to increased temperatures of 200 °C, 400 °C, 600 °C, 800 °C, and 1000 °C. It can be shown that water absorption increases as the temperature of the test specimens exposed to fire rises. It gradually raises the temperature until it reaches 400 °C. At 600 °C, 800 °C, and 1000 °C, greater temperatures result in increased water absorption. Water absorption was measured at 13.3% by weight up to 400 °C for 10% replenishment, suggesting that pores did not expand significantly as the temperature rose. However, at higher temperatures (1000 °C), it increased by approximately three times, indicating fast degradation in all mixtures. This is owing to the development of large voids in both mixtures due to the dehydration of the C-S-H gel [17,28,35,81,82,83,84,85,86,87,88,89,97,98,99,100,101,102,103]. Due to the differences in water absorption of the two distinct materials, concrete mixes comprising marble dust and foundry sand absorb less water than control mixes.

### 3.10. Mass Loss

**Figure 14:**
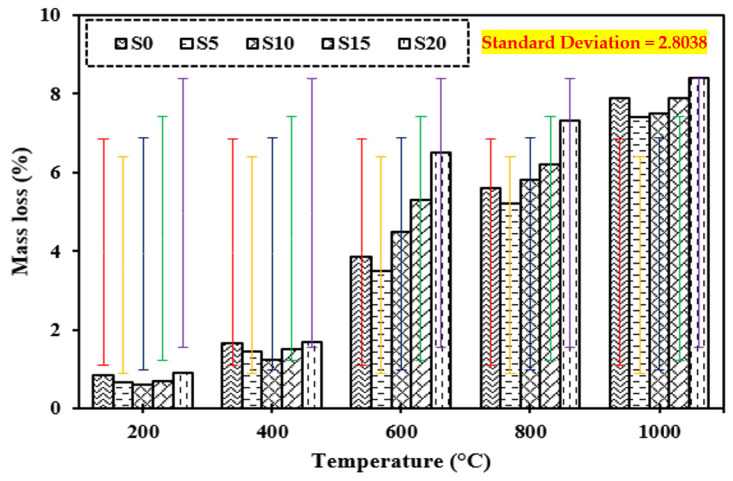
Mass loss of concrete after exposure to elevated temperature.

Figure 14 shows the mass loss of the control mix and concrete including marble dust and foundry sand when subjected to high temperatures of 200 °C, 400 °C, 600 °C, 800 °C, and 1000 °C. It can be shown that as the temperature of the control mix and the concrete containing marble dust and foundry sand is raised, the loss in mass of the concrete increases. The higher the temperature rises; the more concrete mass is lost [17,28,35]. It was negligible up to 400 °C, but increased dramatically after the temperature rose above 600 °C. The mass loss in concrete when exposed to an elevated temperature up to 400 °C was similar to the equivalent cases of the control mix. In both situations, this is due to water loss from the matrix owing to evaporation. Because of the development of voids in the concrete, a higher mass loss of 40.7% was observed for marble dust and foundry sand concrete samples exposed at 600 °C and beyond. Due to the conversion of free moisture to vapor, weight was initially lost. When the temperature was elevated to around 600 °C to 800 °C, the chemically bonded water from the C-S-H gel evaporated, producing fissures on the test objects’ surfaces, as seen in Figure 14. The significant loss in weight of concrete was ascribed to the phase transition of aggregate and dissociation of cement paste at higher temperatures of 1000 °C.

### 3.11. Ultrasonic Pulse Velocity

**Figure 15:**
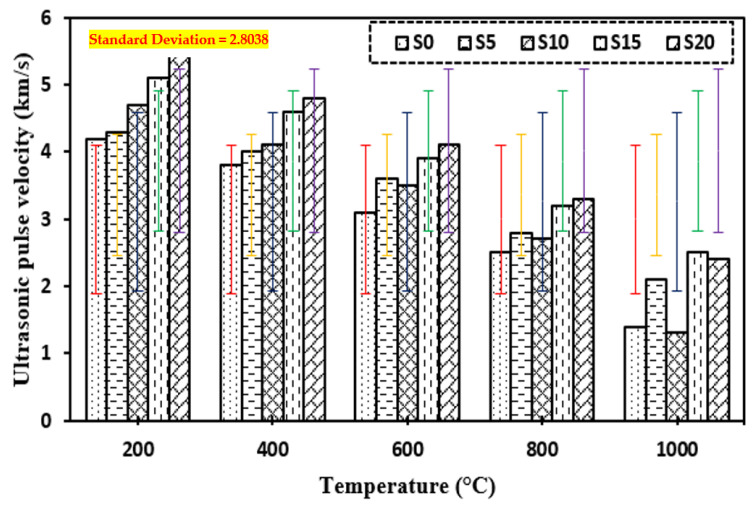
UPV of concrete after exposure to elevated temperature.

Figure 15 shows the ultrasonic pulse velocity of the control mix and concrete including marble dust and foundry sand subjected to higher temperatures of 200 °C, 400 °C, 600 °C, 800 °C, and 1000 °C. The findings of the experiments demonstrate that when the temperature rises, the UPV of concrete mixtures decreases. Temperature increases porosity in concrete mixes, resulting in lower UPV values, although all concrete mixes have UPV values of more than 3.2 km/s, as required by IS 13311 (Part 1): 1992, up to 600 °C. Increased porosity owing to rising temperatures from 200 °C to 1000 °C is the cause of the decrease in UPV of all mixtures. Due to capillary water loss and ettringite dehydration, the temperature dropped dramatically from 400 °C to 1000 °C. Overall, the UPV values for both concrete mixes are adequate up to 600 °C and above the value of 3.2 km/s as specified by IS 13311 (Part 1): 1992.

## 4. Conclusions

Based on the test results for both concrete mixes (with and without replacement), the following conclusions may be drawn:

(i) The compressive strength of concrete samples tested at ambient temperature (29 °C) increased by 10.7% when 10% of the cement was replaced by marble dust and fine sand was replaced by foundry sand, and decreased by 11% when 20% of the cement was replaced by marble dust and fine sand was replaced by foundry sand, and at elevated temperatures increased by 21.5% in the case of annealing and 16.3% in the case of quenching under up to 400 °C elevated temperature on 10% replacement of cement by marble dust and fine sand replaced by foundry sand, and then decreased 54.8% and 52.5% as the temperature increased from 600 °C to 1000 °C in cases of annealing and quenching. This is related to the creation of tiny fractures in both normal and marble dust and foundry sand concrete owing to the breakdown of C-S-H gel along with the voids and empty water capillaries
(ii) The flexural strength of concrete samples tested at elevated temperatures increased by 12.7% with a 5% replacement of marble dust and foundry sand in the case of annealing and 15.6% with a 10% replacement of marble dust and foundry sand in the case of quenching up to 400 °C, and then decreased by 40% and 51.7% with an increase in temperature ranging from 600 °C to 1000 °C elevated temperature in case of annealing and quenching. The rapid drop in flexural strength is attributed to the development of widening fractures around 1000 °C. However, the connection between aggregate and cement pastes is weakened by shrinkage produced by the evaporation of free water and chemically bound water from the cement paste.
(iii) The tensile strength of concrete samples tested at elevated temperatures increased by 31.1% in the case of annealing and 16.9% in the case of quenching under up to 400 °C elevated temperature on 10% replacement of cement by marble dust and fine sand replaced by foundry sand, and then decreased by 52.5% and 53.3% in cases of annealing and quenching as the temperature increased from 600 °C to 1000 °C.
(iv) The percentage reduction in static modulus was increased with elevated temperature; at 1000 °C the reduction was higher for concrete containing marble dust and foundry sand (77.4% for 5% and 86.9% for 20% replacement) than the control mix (72.7%). Similarly, for dynamic modulus the percentage reduction was increased with elevated temperature, at 1000 °C was higher for concrete containing marble dust and foundry sand (76% for 5% and 94.1% for 20% replacement) than for the control mix (81.5%). The larger decrease can be ascribed to the decomposition of marble dust and foundry sand, which causes cracks and voids in the cement paste matrix, causing the concrete structure to weaken.
(v) Water absorption was measured at 13.3% by weight up to 400 °C for 10% replacement, suggesting that pores did not expand significantly as the temperature rose. However, at higher temperatures (1000 °C), it increased by approximately three times, indicating fast degradation in all mixtures.
(vi) The mass loss of concrete containing marble dust and foundry sand was similar to the control mix when subjected to a temperature up to 400 °C. In both situations, this is due to water loss from the matrix owing to evaporation. Because of the development of voids in concrete, a higher mass loss of 40.7% was observed for marble dust and foundry sand concrete samples exposed at 600 °C and beyond.
(vii) Although all concrete mixes have UPV values of more than 3.2 km/s, as specified by IS 13311 (Part 1): 1992, up to 600 °C, temperature increases porosity in concrete mixes, resulting in lower UPV values.

According to the results of the experiments presented in this study, substituting up to 10% of marble dust and foundry sand had no effect on the residual properties of concrete when subjected to temperatures up to 400 °C. However, at temperatures above 400 °C, the degradation of concrete was observed, resulting in a rapid reduction in the residual properties of concrete, mainly for replacement of more than 10% of the mixture.

## Figures and Tables

**Table 1:** Chemical properties of cement.

Ingredient	CaO	SiO_2_	Al_2_O_3_	Fe_2_O_3_	MgO	SO_3_	K_2_O	LOI
**Percentage**	60.5	22.5	4.2	3.6	2.7	2.3	0.6	1.87

**Table 2:** Chemical composition of marble dust.

Ingredient	CaO	SiO_2_	Al_2_O_3_	Fe_2_O_3_	MgO
**Percentage**	40.45	28.35	0.42	9.70	16.25

**Table 3:** Chemical composition of foundry sand.

Ingredient	SiO_2_	Fe_2_O_3_	Al_2_O_3_	CaO	MgO	TiO_2_	Na_2_O	K_2_O	SO_3_	Mn_3_O_4_
**Percentage**	83.8	5.39	0.81	1.42	0.86	0.22	0.87	1.14	0.21	0.047

**Table 4:** Ratios of concrete mixed with marble dust and foundry sand.

Samples	Cement (kg/m^3^)	Marble Dust (kg/m^3^)	Fine Aggregate (kg/m^3^)	Foundry Sand (kg/m^3^)	Coarse Aggregate (kg/m^3^)	Admixture (%)	Water (kg/m^3^)	Slump (mm)
S0	407.07	0	851.15	0	1083	0.6	162.82	75
S5	386.72	20.35	808.59	42.55	1083	0.6	162.82	80
S10	366.36	40.71	766.03	85.12	1083	0.8	162.82	90
S15	346.01	61.06	723.48	127.67	1083	0.8	162.82	95
S20	325.66	81.41	680.92	170.23	1083	0.8	162.82	108

## Data Availability

No data were used to support this study.
